# Yeast Immobilization Systems for Alcoholic Wine Fermentations: Actual Trends and Future Perspectives

**DOI:** 10.3389/fmicb.2018.00241

**Published:** 2018-02-15

**Authors:** Jaime Moreno-García, Teresa García-Martínez, Juan C. Mauricio, Juan Moreno

**Affiliations:** ^1^Department of Microbiology, Agrifood Campus of International Excellence (ceiA3), Campus de Rabanales, University of Cordoba, Cordoba, Spain; ^2^Department of Agricultural Chemistry and Soil Science, Agrifood Campus of International Excellence (ceiA3), Campus de Rabanales, University of Cordoba, Cordoba, Spain

**Keywords:** yeast immobilization, wine, yeast biocapsules, fermentation, yeast metabolism

## Abstract

Yeast immobilization is defined as the physical confinement of intact cells to a region of space with conservation of biological activity. The use of these methodologies for alcoholic fermentation (AF) offers many advantages over the use of the conventional free yeast cell method and different immobilization systems have been proposed so far for different applications, like winemaking. The most studied methods for yeast immobilization include the use of natural supports (e.g., fruit pieces), organic supports (e.g., alginate), inorganic (e.g., porous ceramics), membrane systems, and multi-functional agents. Some advantages of the yeast-immobilization systems include: high cell densities, product yield improvement, lowered risk of microbial contamination, better control and reproducibility of the processes, as well as reuse of the immobilization system for batch fermentations and continuous fermentation technologies. However, these methods have some consequences on the behavior of the yeasts, affecting the final products of the fermentative metabolism. This review compiles current information about cell immobilizer requirements for winemaking purposes, the immobilization methods applied to the production of fermented beverages to date, and yeast physiological consequences of immobilization strategies. Finally, a recent inter-species immobilization methodology has been revised, where yeast cells are attached to the hyphae of a Generally Recognized As Safe fungus and remain adhered following loss of viability of the fungus. The bio-capsules formed with this method open new and promising strategies for alcoholic beverage production (wine and low ethanol content beverages).

## Introduction

Yeast immobilization offers numerous opportunities for industrial fermentation processes such as beer, cider production, or winemaking. This technology aims to confine intact, active yeast cells to a specific region, thus increasing the cell density, permitting the enhancement and prolongation of certain metabolites (e.g., aromatic) production, allowing better control and stability of the yeast strain, providing cell protection against shear forces, and enabling cell recovery/reutilization and continuous fermentations, among other advantages (Williams and Munnecke, 1981; Groboillot et al., 1994; Sakurai et al., 2000; Kourkoutas et al., 2004b; Baptista et al., 2006; Nedović et al., 2015).

Although this technology reduces process cost and allows customization of wine properties, industrial use of immobilized cells is still limited (Djordjevic et al., 2016; Berbegal et al., 2017). Nedović et al. (2015) proposed that future investigation should approach the storage of immobilized cells long-term and new designs of the processes and bioreactors that are simple, flexible, and non-expensive and can be readily scaled up. Moreover, to accomplish crucial factors in winemaking like consumer acceptance, safety issues, and profitability, Kourkoutas et al. (2004b) recommended supports that are abundant in nature, cost-effective, and of food-grade quality for successful industrial application. Nevertheless, questions such as “what particular immobilization system utilize in what wine elaboration process” or “how immobilization affects cell physiology, flavor formation, and wine stability – including microbial, chemical, and sensorial” still need to be addressed in order to promote yeast immobilization technologies in wine industrial processes.

The overall objective of this review is to compile the most updated information about the requirements of cell immobilizers for winemaking, the immobilization systems applied and proposed to the production of wine (including advantages and drawbacks), and yeast physiological consequences of immobilization strategies. Special attention was placed on inter-species immobilization methodologies, which are considered novel approaches for winemaking and other fields of applications. This is the case for “yeast biocapsules” which consist of yeast cells attached to the hypha of a dead filamentous fungus cataloged as Generally Recognized As Safe (GRAS).

## Cell Immobilizer Requirements for Winemaking Purposes

Accurate selection of the immobilization method and the carrier material (with consideration of legality and stability, safety, operating costs, and product quality) is essential. Among the production systems that have been the subject of investigations, some seem to fulfill the above prerequisites and lead to promotion of aroma formation during the fermentation process and improvement of the overall sensory characteristics of the final products, e.g., wine, beer, and cider. However, actions should be also focused on economical, abundant, non-damaging, and food-grade immobilization supports, which will ameliorate quality and provide a singular aroma profile and fine taste to the final product. In general, for alcoholic beverage production purposes, the cell carrier has to comply with certain requirements as follows (Martin and Etievant, 1991):

(i) Big surface, with functional properties and/or chemical groups favoring cells to adhere.
(ii) Easy to handle and regenerate.
(iii) High and retained cell viability and operational stability.
(iv) Catalytic activity not affected.
(v) Uniform and controllable porosity to allow free exchange of substrates, products, cofactors, and gases.
(vi) Good mechanical, chemical, thermal, and biological stability.
(vii) Easy, cost-effective, and amenable to scale-up immobilization technique.
(viii) Not affect product quality.

## Immobilization Methods Depending on the Yeast Cell Localization

**FIGURE 1:**
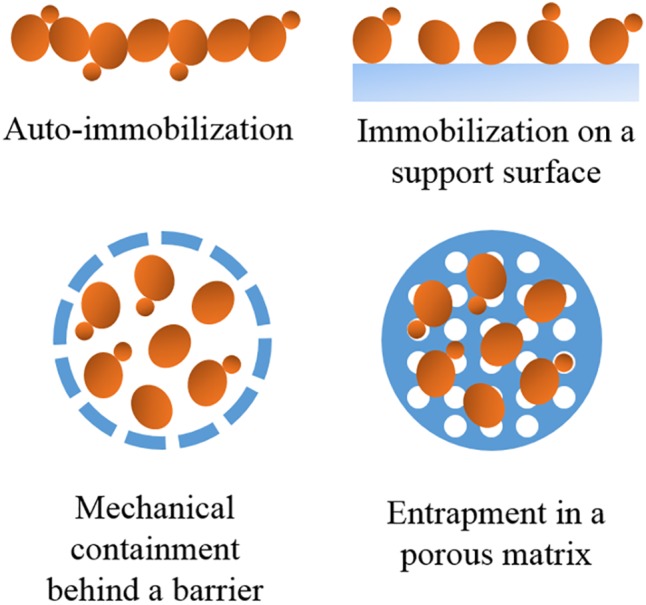
Basic methods of cell immobilization depending on the cell localization.

To date, different methods for yeast immobilization have been developed depending on the mechanism of cell localization (**Figure 1** and **Table 1**).

**Table 1:** Methods of yeast immobilization: brief description, advantages, disadvantages, and examples of applications in winemaking.

Methods of immobilization	Brief description	Advantages	Disadvantages	Examples in winemaking (proposed or industrially applied)
Auto-immobilization	Innate ability of cells to aggregate (i.e., adhesion, biofilm formation, filament formation, and flocculation).	Beneficial effects on wine quality and industrially used.	Sensitive to factors like pH, medium, composition, O_2_ content, etc.	Biofilms of flor yeasts are traditionally used in biological aging for Sherry wine elaboration. Biofilms were also proposed for the reduction of the ethanol content in wines (Moreno et al., 2016). Flocculation is being used for wine clarification in sparkling wine production.
Immobilization on a support surface	Adsorption of cells to a carrier by cell membrane-immobilizer covalent bonding or by electrostatic forces.	Cheap carrier materials and ease of carrying out the process.	Depth and bonding strength of the cells are not determined. Potential detachment of yeast cells.	Cellulose covered with Ca-alginate and DEAE-cellulose covered with an anion-exchange resin were recommended by Lommi and Advenainen (1990) while gluten pellets and delignified cellulosic materials were recommended for room and low-temperature industrial fermentations (Bardi and Koutinas, 1994; Bardi et al., 1996a,b, 1997; Mallouchos et al., 2003). Fruit pieces were investigated for continuous processes (Kourkoutas et al., 2001, 2002, 2003a,b), and combined alcoholic-malolactic fermentations (Mallouchos et al., 2002; Genisheva et al., 2014).
Mechanical containment behind a barrier	Cells are entrapped in microporous or ultraporous membrane filters, microcapsules or on an interaction surface of two immiscible liquids.	Useful when minimal transfer of compounds or cell-free products is needed.	Cell loss during mass transfer and possible membrane biofouling.	Glass pellets coated with a membrane of alginates were proposed for batch and continuous winemaking processes (Ogbonna et al., 1989), “Millispark” cartridge for sparkling wine production (Ramon-Portugal et al., 2003) and a two-vessel bioreactor system (one operated as a membrane bioreactor) employed for continuous dry winemaking (Takaya et al., 2002).
Entrapment in a porous matrix	Cells incorporation to rigid networks.	Prevention of cell diffusion and allowance of transfer of substrates and metabolism products.	High cost, low mechanical, and chemical stability. The biomass entrapped in a gel matrix is critical for usage of biotechnological processes utilizing viable immobilized yeast cells.	Ca-alginate gels were promoted for clarification in sparkling winemaking (Colagrande et al., 1994; Fumi et al., 1988), cell-recycle batch process and optimization of primary must fermentations (Suzzi et al., 1996), increase glycerol of wines (Ciani and Ferraro, 1996; Ferraro et al., 2000), treatment of sluggish and stuck fermentations (Silva et al., 2002), removal of ethanol or toxins (Canonico et al., 2016; Farbo et al., 2016), and simultaneous alcoholic-malolactic wine fermentations (Bleve et al., 2016).
Natural supports	Principle of food-grade purity and used with slightest or no pre-treatment.	High abundance, low cost, and food-grade nature.	Degradation process of the supports not evaluated. Industrial scale-up not described.	Delignified cellulosic material, gluten pellets, grains, and fruit pieces were proved effectively for winemaking. Yeast immobilized in a GRAS fungi (yeast biocapsules) has been tested for white wine, sparkling wine, and natural sweet wine elaboration (Peinado et al., 2004; Puig-Pujol et al., 2013; García-Martínez et al., 2015). Usage of corn grains for ambient/low temperature batch fermentations was found adequate (Kandylis et al., 2012). Delignified cellulosic material was proven for simultaneously alcoholic-malolactic fermentations (Servetas et al., 2013).
Organic supports	Synthetically made (e.g., plastic) or extracted from natural sources by more complex processes (e.g., polymeric hydrogels) regardless of their food-grade purity.	Ability to gel under mild conditions and form spherical beads that protects against contamination and inhibitory substances while favoring substrate utilization and enhancing stability, flavor productivity and efficiency.	High costs, low mechanical, and chemical stability.	Alginate gels have been commercially applied for sparkling wine production (Fumi et al., 1988; Busova et al., 1994; Colagrande et al., 1994). Organic supports have been also successfully applied to: continuous fermentations (Ogbonna et al., 1989), pomegranate winemaking at ambient temperatures (Sevda and Rodrigues, 2011), wine produced from the tropical fruit cagaita (Oliveira et al., 2011), wine from cabernet sauvignon and pinot noir grape varieties (Andrade Neves et al., 2014).
Inorganic supports	Not organic materials like porous ceramics, porous glass, polyurethane foam, etc.	Usually abundant and can improve fermentation productivity and aroma.	Strong changes in cell metabolism and viability. High concentrations of mineral residues.	Mineral kissiris proposed in low temperature winemaking (Bakoyianis et al., 1992, 1993), γ-alumina and kissiris for continuous or batch alcoholic fermentations (Kana et al., 1992; Loukatos et al., 2000). Gellan particles cross-linked with magnesium acetate were proposed for alcoholic fermentation of grape must (Iurciuc et al., 2016).

### Auto-Immobilization

Winemakers benefit from the ability of certain yeasts species to auto-immobilize in an innate way. From a biological point of view, immobilization favors yeast cells as it allows cell cooperation to fully utilize available resources and maximize chances of survival through improved resistance to stress (Honigberg, 2011). Microorganisms, notably *Saccharomyces cerevisiae* can perform various multi-cellular manners of immobilization: adhesion, biofilm formation, filament formation, and flocculation. The effect of some of these behaviors on the wine quality is widely known to be beneficial and is already applied industrially. This is the case of yeast biofilm formation for biological aging in the elaboration of Sherry wines and flocculation for the second fermentation of sparkling wines.

Yeast immobilization in biofilms is formed spontaneously in the wine-air interface of wines that are stored in barrels during a process that is known as “biological aging.” This type of biofilm is called “flor” or “velum” – formed by special yeast strains known as “flor yeasts” – and protects wine from oxidation and influences the sensory properties of Sherry type wines. The yeast metabolic activity mainly results in a consumption of ethanol and glycerol – the major carbon sources – and production of acetaldehyde – the main metabolite liberated into the aged wine. Additionally, consumption of ethanol raises the contents of acetic acid, acetoin, and 2,3-butanediol and promotes their inclusion as carbohydrates, lipids, and proteins into yeast cells via the Krebs Cycle (Martínez et al., 1998; Zara et al., 2010; Moreno and Peinado, 2012; Moreno-García et al., 2013, 2014, 2015a,b, 2017). The resulting wines are characterized by sensorial characteristics known as flor or velum bouquet (López-Alejandre, 2005).

Cell flocculation consists of non-sexual aggregation of single-celled organisms in suspension to form a larger unit or aggregates of many cells known as flocs (Jin and Speers, 1998). The large size of the flocs makes their potential use in reactors feasible. It is considered the simplest and cheapest immobilization technique although it is easily influenced by several factors like cell wall composition, medium, pH, and dissolved oxygen (Kourkoutas et al., 2004b; Nedović et al., 2005). It is used in the production of sparkling wines, such as Champagne, performed by the “Champenoise” technique, which consists of a second fermentation in a sealed bottle of a base wine previously obtained by fermentation of a grape must. In the last phase of this course, the bottles are turned down and yeast cells deposit on the neck of the bottle. Here, the utilization of flocculent yeast cells is important as it eases the process of removing cell deposit from the bottle, clarifying the wine, and reducing wine losses (a process called dégorgement) (Valles et al., 2008). Simultaneously, yeast immobilization through flocculation reduces the wine production costs as there is less energy expended, thus turning into a ‘greener’ process that could enhance the quality of final products. It is also used in the brewing industry as packed-bed or fluidized-bed or even continuous stirred-tank reactors (Kourkoutas et al., 2004b) and it affects fermentation productivity and quality, as well as yeast removal and retrieval. Agents or cross-linkers can enhance flocculation of cells that do not spontaneously aggregate.

### Immobilization on a Support Surface

Immobilization on a support surface is defined as the binding of yeast cells to a carrier by covalent bonding between the cell and the support, or by adsorption (ionic bonds or electrostatic forces). Examples of known support surfaces are cellulosic materials like diethylaminoethyl-cellulose (DEAE-cellulose), delignified sawdust, sawdust, and wood; or inorganic materials like hydromica, montmorillonite, palygorskite, porous glass, and porous porcelain. This method has been widely applied due to low cost of used immobilization materials, such as cellulosic and inorganic materials, and the simplicity of achieving the process. However, the depth of the cell biofilm and the bonding strength often vary and are not readily determined. As cells are directly exposed to the solution, detachment and relocation are possible while yeast growth.

Among the cellulosic material, fruit pieces, delignified cellulosic materials (DCMs), and gluten pellets (GPs) have been applied in winemaking. Fruit pieces ease the immobilization methods required. Apple and quince constitute abundant and low price supports of food-grade purity of immobilization that were found suitable for continuous processes and lead to production of improved sensory traits (Kourkoutas et al., 2001, 2002, 2003a,b). Further, grape skins were used to immobilize *S. cerevisiae* because of easy application, increased productivity, and positive influence on wine aroma compared to free cells (Mallouchos et al., 2002). This support was established by these authors as suitable for winemaking and proposed for future investigation to their utilization in combined alcoholic fermentation (AF) and malolactic fermentations (MLFs). On the other hand, DCM and GP were considered effective in carrying out fermentations at both room- and low-temperature as well as increasing rates and improving organoleptic quality compared to free cells (Bardi and Koutinas, 1994; Bardi et al., 1996a,b, 1997; Mallouchos et al., 2003). DCM and GP were proposed to use at industrial levels because they are inexpensive and abundant supports of food-grade purity that are easy to produce industrially. In comparison with other natural supports, they lower fermentation rates, present a longer operational stability, are suitable for low-temperature winemaking, and also accepted by consumers. Yeast cells immobilized with DCM and GP were found to fit commercialization objectives through freeze-drying techniques as the freeze-dried immobilized yeasts produced wines of similar quality to those made by fresh immobilized yeast cells and of enhanced properties in comparison with free cells (Iconomopoulou et al., 2000, 2002, 2003; Bekatorou et al., 2001). This last feature makes DCM and GP attractive for industrial use.

Inorganic support surfaces (e.g., palygorskite, hydromica, and porous porcelain) were shown to have mainly few advantages in winemaking (Hamdy et al., 1990; Colagrande et al., 1994). Researchers recommended the utilization of cellulose and DEAE-cellulose (as main carrier) covered with Ca-alginate and an anion-exchange resin, respectively, as immobilization supports for winemaking; the first for continuous winemaking purposes (Lommi and Advenainen, 1990). Increases of calcium ion contents and off-flavors due to the use of alginate or DEAE-cellulose, respectively, must be considered in winemaking processes.

### Mechanical Containment behind a Barrier

The most common are the microporous or ultraporous membrane filters and the microcapsules. They are utilized when the minimal transfer of compounds or cell-free products is necessary (Park and Chang, 2000). This cell immobilization type can be attained by three methods: (i) by utilization of microporous membrane filters, (ii) by entrapment of cells in a microcapsule, or (iii) by cell immobilization on to an interaction surface of two immiscible liquids. It has been used in winemaking, and however, its use is limited because of loss during mass transfer (Lebeau et al., 1998) and potential membrane biofouling caused by cell growth (Gryta, 2002). “Millispark” cartridge developed by Millipore is an example utilized for secondary fermentation in a bottle of sparkling winemaking (Ramon-Portugal et al., 2003). *S. cerevisiae* and *Schizosaccharomyces pombe* were co-immobilized on glass pellets coated with a membrane of alginates to further use them for batch and continuous winemaking processes (Ogbonna et al., 1989). Wines with similar features to those produced with free cells were obtained. Takaya et al. (2002) reported that a system consisting of two-vessel bioreactor (one operating as a continuous stirred tank reactor and the other one as the membrane bioreactor), where cells were entrapped by a cross-flow type microfilter, was suitable for continuous dry winemaking and had 28-fold higher production than a batch system. Moreover, microfiltration and ultrafiltration membranes as well as silicon, ceramic, and other membranes have been employed.

### Entrapment in a Porous Matrix

Entrapment in a porous matrix is attained when cells are incorporated in a rigid network which prevents them from diffusing into the neighboring medium while still admitting mass transfer of substrates and metabolism products. They are divided in two methods: (i) cells infiltrate into the porous matrix until their motility is interfered by other cells and (ii) the porous material is assembled *in situ* into a culture of cells. Some examples are polysaccharide gels like alginates, *k*-carrageenan, agar, chitosan, and polygalacturonic acid or other polymeric matrixes like gelatine, collagen, and polyvinyl alcohol (Norton and D’Amore, 1994; Park and Chang, 2000). One of the problems of this technique is cell release when located on the outer surface of the matrix. To bypass this possibility, double layer beads have been used (Tanaka et al., 1989; Taillandier et al., 1994; Ramon-Portugal et al., 2003). In general, the use of polysaccharide hydrogels and alginates is not a suitable industrial choice for several reasons: (i) high prices, (ii) low mechanical and chemical stability that causes cell and residues release in wine, and (iii) biomass entrapped in a gel matrix that is critical for utilization of biotechnological processes using viable immobilized cells (Kourkoutas et al., 2004b).

Salts like Na-, Ca-, or Ba-alginate have been extensively used for cell immobilization, and among them, Ca-alginate gels are the most advisable for AF (Colagrande et al., 1994). Notably, Ciani and Ferraro (1996) proposed a system entrapping *Candida stellata* in Ca-alginate gels as an attractive system to increase glycerol content in wine. These authors reported a 30-fold improvement in fermentation rate (g of CO_2_/day) in comparison with free cells and twofold production of ethanol and a reduction in acetaldehyde and acetoin production. Moreover, Ferraro et al. (2000) attempted to scale up the immobilization systems to pilot and industrial scales. They revealed an interesting flavor profile of wines produced when co-inoculated with *S. cerevisiae*, and however, the wild wine microbiota was not completely repressed. Ca-alginate beads have also been recommended to entrap highly flocculent *S. cerevisiae* strains to perform cell-recycle batch process and optimize primary must fermentations (Suzzi et al., 1996). Another application of Ca-alginate cell entrapment is the secondary fermentation in sparkling winemaking for easy clarification and removal of cells. *S. cerevisiae* strains are being immobilized for this purpose and commercially applied in winemaking processes (Fumi et al., 1988; Colagrande et al., 1994). *S. cerevisiae* encapsulated in Ca-alginate were also utilized with success for the treatment of sluggish and stuck fermentations and revealed better results than the traditional free cells method – the system attained a decrease rate of 2.8 g/L × day of reducing sugar with a viable cell concentration of 5 × 10^6^/mL and no increase in off-flavor content or volatile acidity (Silva et al., 2002). According to the winemakers, one of the major drawbacks of calcium salt-based systems is the high Ca^2+^ content provoked by the low solubilization of calcium tartrate in the bottled wine.

## Immobilization Methods Depending on the Chemical Composition of the Carrier

The materials used as immobilization supports (carriers), can be divided, based on their origin, into natural materials and artificially treated materials; according to Kourkoutas et al. (2010) and Nedović et al. (2010), they can be categorized as shown in **Table 1**.

### Natural Supports

Carrier materials are mainly of food-grade purity and are used with minimal to no pre-treatment; like brewer’s spent grains, DCM, GP, pieces of fruit, sawdust, wood, etc. Their abundance, low cost and food-grade composition have made them an interesting way to enhance the aroma character of many products, e.g., wine, beer. The utilization of natural supports such as DCM, GP, grains, and fruit pieces, for immobilization, was proved effective for winemaking as previously mentioned (see the section “Immobilization on a Support Surface”). Natural materials with certain food-grade meet the prerequisites for the selection of the carrier and result in promoting aroma formation and advancement of the sensory features of the final fermented product. *S. cerevisiae* cells immobilized in corn grains were considered a good candidate system because it was efficient for fermentations at ambient and low temperatures during repeated batch fermentations of grape must (Kandylis et al., 2012).

### Organic Supports

Organic supports are artificially made (e.g., plastic) or obtained from natural sources by more complicated techniques (e.g., polymeric hydrogels) regardless of their food-grade composition. Natural or synthetic polymers have been widely researched most probably due to their gel-forming ability under gentle conditions and the capacity to form spherical beads that protect yeast cells against contamination and inhibitory substances while favoring substrate utilization and improving stability, flavor production, and efficiency (Nedović et al., 2010). Most used are those comprised of alginates, cellulose, carrageenan, agar, pectic acid, and chitosan. Ca-alginate gels among them are more convenient for AF (Colagrande et al., 1994), and however, the use of alginates and polysaccharide hydrogels generally did not offer a favorable industrial alternative as previously explained (see the section “Entrapment in a Porous Matrix”). Most attempts were made for the utilization of alginate gels for the second fermentation in order to improve the technology of sparkling wine and have been commercially applied (Busova et al., 1994; Colagrande et al., 1994; Fumi et al., 1988). Immobilization of yeasts in organic supports has also been successfully applied to the following: mead production (Pereira et al., 2014), pomegranate winemaking (Sevda and Rodrigues, 2011), wine made from the tropical fruit cagaita (Oliveira et al., 2011), wine from Cabernet Sauvignon or Pinot noir grape varieties (Andrade Neves et al., 2014), green beer production (Wang et al., 1989), stout beer production (Almonacid et al., 2012), lager-beer (Naydenova et al., 2013), and cider (Nedovic et al., 2000).

### Inorganic Supports

Several inorganic materials such as porous ceramics, porous glass, polyurethane foam, etc., have been introduced as yeast cell carrier materials for many fermentation processes: beer production (Virkajärvi and Pohjala, 2000; Virkajärvi et al., 2002; Kourkoutas et al., 2004b) and wines (Ogbonna et al., 1989; Bakoyianis et al., 1992, 1993; Kana et al., 1992; Loukatos et al., 2000; Bonin and Skwira, 2008). However, even though they are usually abundant and can improve fermentation productivity and aroma, they can experience strong changes in metabolism and viability as the cells used in artificial immobilization methods are not in their natural form. Also, they are usually considered undesirable for winemaking due to high concentrations of mineral residues found in the product. Nonetheless, their use in immobilization systems can be regarded as promising for use in bioethanol or distillates production.

### Other Materials

Other methods of immobilization such as membrane systems, entrapment by various types of interaction (i.e., Van der Waals’ forces, ionic bonds, hydrogen bridges) and multi-functional agents – several functionalities integrated into a single miniaturized device (i.e., glutaraldehyde-based system) – are scarcely treated. As earlier cited, Takaya et al. (2002) revealed a membrane-based bioreactor as a good candidate system for continuous dry wine production. Ligno-cellulosic materials from agricultural wastes can be valuable substrates for immobilization, after removal of the lignin fraction from the cellulose matrix by an alkaline treatment in view to create tubular cellulose–based (TC) nanoestructures.

## Yeast Physiological Consequences of Immobilization Strategies

Cell growth, physiology, and metabolic alterations may be induced by immobilization although they are hard to predict (Melzoch et al., 1994; Norton and D’Amore, 1994; Walsh and Malone, 1995; Djordjevic et al., 2016).

Assays comparing immobilized and free cells have revealed effects on increase in stored polysaccharides, altered growth rates, lower yield of fermentation by-products, activation of yeast energetic metabolism, increased substrate uptake and product yield, higher intracellular pH, increased resistance against toxic and inhibitory compounds, and increased invertase activity (Norton and D’Amore, 1994). Immobilization of yeast to various solid surfaces affects intrinsic cell growth rate, which either increased (Bandyopadhyay and Ghose, 1982) or decreased (Doran and Bailey, 1986). The pH in immobilized *S. cerevisiae* cells in alginate beads is lower than in free yeasts, 6.8 and 6.9, respectively, which was attributed to increased permeability of the cell membrane for protons, leading to a higher ATP utilization and activating glycolysis and glucose uptake (Galazzo and Bailey, 1990). This results in an increased enzyme activity and thus more substrate channeled to biomass and ethanol production. Norton and D’Amore (1994) reported an enhanced ethanol resistance, a partial removal of substrate inhibition by cell immobilization, and higher tolerance to toxic compounds. These authors suggested that an increased ethanol tolerance might be due to a modification in concentration of membrane fatty acid because of oxygen diffusion limitations or simply due to cell encapsulation by a protective layer of the immobilization material. On the other hand, the tolerance to toxic compounds can be indirectly related to osmotic stress that leads to intracellular production of compounds like polyols that regulate pressure, which also leads to diminished water activity and consequently increased tolerance to toxic chemicals (Norton and D’Amore, 1994). Finally, Lodato et al. (1999) showed higher thermal stability in immobilized yeasts.

In immobilized cell fermentations, increased ester and decreased fusel alcohols formation and the ratio of esters to alcohols have the highest influence on beverage technology (Bardi and Koutinas, 1994; Mallouchos et al., 2003). Some trials have been attempted to model the accumulation of dominant yeast metabolites produced by free and immobilized cells (Vassilev et al., 2013). Nagarajana et al. (2014) observed a permanent pattern of gene expression different from starving planktonic cells: highly expressing genes in cell wall reassembling and stress tolerance, glycolysis, but decreasing transcription of genes that regulate the cell cycle and in the tricarboxylic acid cycle. Consequently, changes in concentrations of metabolites are observed when using entrapped or adsorbed yeast cells. Special attention has to be given to compounds such as alcohols (ethanol, higher alcohols), carbonyl compounds (acetaldehyde, vicinal diketones), esters (acetate esters, medium-chain fatty acid esters), organic acids (medium-chain fatty acids), and sulfur compounds (hydrogen sulfide, sulfur dioxide, dimethyl sulfide) as they are those that most affect flavor during fermentation (Dufour et al., 2003; Nedović et al., 2015). In fermented beverages, the greatest aroma impact is due to increased esters, decrease of fusel alcohol concentrations, and ratio of esters/alcohols from fermenting yeast metabolism (Bardi and Koutinas, 1994; Mallouchos et al., 2003; Nedović et al., 2015). In white wine production, it was detected a significant difference in sensory properties among free and immobilized cells (Mallios et al., 2004; Tsakiris et al., 2004a,b; Genisheva et al., 2012). Kourkoutas et al. (2004b) noted a stronger flavor and aroma in semi-sweet wines when immobilizing yeasts. Kourkoutas et al. (2004a), Tsakiris et al. (2004a,b), and Gonzalez-Pombo et al. (2014) did not report an important influence on the pleasantness of wine. A slight difference was revealed in the scores for preference of the produced wines, where scores were higher when using immobilized cells compared to free cells (Tsakiris et al., 2004a,b). These authors also found that temperature is an important factor for wines elaborated with immobilized yeasts at lower temperatures, which were preferred by the consumers. Another aim of yeast immobilization in winemaking is the removal of the off-flavor aroma compounds, as well as the de-acidification of wines to enhance the organoleptic features of the final product (Genisheva et al., 2012, 2013, 2014; Vilela et al., 2013). Nedović et al. (2015) suggested more attention to be placed on evaluation of sensory quality of wine produced by free and immobilized cells with a trained panel or consumers along with instrumental analyses in order to assess the quality of final products and further support the development of acceptable products before being marketed.

## New Trends of Yeast Immobilization in Winemaking

During the last few years, novel yeast cell immobilization systems have been designed to adapt to winemaking purposes. New materials such as spherical gellan particles cross-linked with magnesium acetate were found suitable for AF of grape must (Iurciuc et al., 2016). In this way, Kumar et al. (2016) studied the use of nano-/micro-porous of cellulosic materials (TC) produced by delignification of mango (*Mangifera indica* L.), sal (*Shorea robusta* G.) sawdust and rice husk (*Oryza sativa* L.) in various food bioprocesses. They conclude that the porous structure of TC renders it suitable for use as carrier for yeast immobilization in AF and also as filter material in microorganism removal processes. The TCs used as *S. cerevisiae* cells immobilization carriers for AF of grape must and glucose media at 15°C provide satisfying fermentation rates, high ethanol content and productivity, and volatile by-products production. In addition, advanced applications in winemaking and co-immobilization of different organisms in different carriers, same carrier, or among each other (exploiting adherence properties of organisms) were recently described.

Canonico et al. (2016) co-immobilized non-*Saccharomyces* yeasts in Ca-alginate to perform sequential fermentations coupled with a final inoculation of free *S. cerevisiae* cells to reduce ethanol content in wine. The yeasts immobilized were Crabtree negative (sugar consumption by respiration and low ethanol yield) and naturally present on grapes and winemaking equipment. The strategy resulted in high reaction rates, avoidance of contamination where the sugar content was reduced to a 50% in 3 days and the ethanol up to 1.6% v/v, and in less prolonged time than in non-immobilized formats. An enhancement of the analytical profile of wine was observed for most of the yeasts immobilized. Although this produced promising results, Canonico et al. (2016) recommended further research because the system submitted uses high inoculation levels and expensive immobilization procedures, which increases the costs of the fermentation process. Then, Moreno et al. (2016) proposed to use the *S. cerevisiae* ability to auto-immobilize in biofilms (i.e., flor velum) to further consume ethanol from a red wine. In this work, the authors observed a decrease in the ethanol content (also 1.6% v/v) and volatile acidity, favorable effect on the color and astringency, and differences in the content of 1-propanol, isobutanol, acetaldehyde, 1,1-diethoxiethane, and ethyl lactate after a short time (40 days) under velum aging conditions. From a sensory analysis, wines were well accepted by the younger consumers in a panel thus, concluding that flor yeasts auto-immobilization in form of biofilms can be used as fining agents supporting new perspectives for the elaboration of new wine types in an inexpensive manner. Another application of yeast immobilization (*Candida intermedia* yeast cells encapsulated in Ca-alginate and magnetic Ca-alginate beads) was discovered by Farbo et al. (2016) objectified to remove the mycotoxin ochratoxin A from rotten grape juice. Although they obtained significant reductions of over 80%, these authors observed a slow release of the mycotoxin by the yeast carriers.

By using the same carrier, *S. cerevisiae* and lactic acid bacteria *Oenococcus oeni* were confined to operate simultaneously AF and MLF with the purpose of enhancing safety and quality of wine (Servetas et al., 2013; Bleve et al., 2016). In this way, Servetas et al. (2013) co-immobilized *S. cerevisiae* and *O. oeni* in DCM carriers covered with starch gel composite and observed a high efficiency at low temperature fermentations (10°C) obtaining a wine characterized by an increased ester formation and lower higher alcohols. Bleve et al. (2016) co-immobilized the same microorganisms in Ca-alginate beads revealing an efficient performance of Negroamaro must fermentation with a decrease of the time needed to complete AF and MLF, low production of volatile acidity and similar organoleptic traits of the wine obtained than with those using sequential AF-MLF in free cell formats. The yeast and bacteria cells immobilized were reused up to three times with no activity loss. Also, *S. cerevisiae* and *O. oeni* entrapped cells into grape stems/skins were used in sequential AF and MLF obtaining wines with sweet and fruity flavors (Genisheva et al., 2014). Nevertheless, it must be considered that simultaneous AF and MLF could also have severe drawbacks sometimes leading to spoilage wines.

**FIGURE 2:**
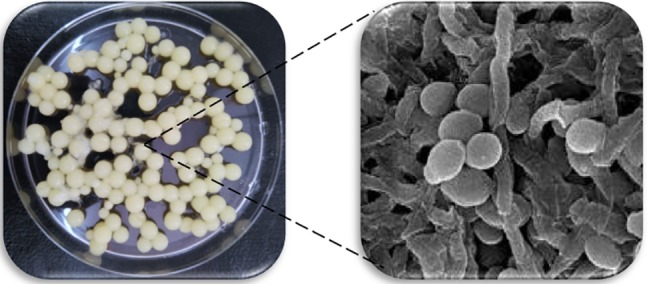
Yeast biocapsules (3–5 mm in diameter) formed with *Saccharomyces cerevisiae* and *Penicillium chrysogenum* **(Left)** and section of the inner wall of a fresh biocapsule exhibiting intact hyphae (filamentous form) with yeast cells (oval shape) photographed in a scanning electron microscope SEM **(Right)**.

Novel concepts of organism co-immobilization without the need of an external support have arose. This kind of methodology exploits the ability of the organisms used to adhere to external bodies. This is the case of the co-immobilization of yeasts and filamentous fungus categorized as GRAS. It consists of the attachment of yeast cells to the mycelium of filamentous fungus (e.g., *Rhizopus* sp., *Aspergillus niger*, and *Penicillium* sp.) (Peinado et al., 2004, 2005, 2006; Nyman et al., 2013) that can be regarded as a natural immobilization system matrix and complies with several required features for the promotion of industrial application: abundant, cheap, storable for long-terms, non-destructive, food-grade, etc. Co-immobilizing *Penicillium chrysogenum* and yeast cells results in the formation of spherical bodies that are hollow, known as “yeast biocapsules” (**Figure 2**). The system minimizes changes to the yeast metabolism and/or yeast viability and enables diffusion of nutrients/products to and from the biocapsules due to the porous structure of the hypha framework (García-Martínez et al., 2011). The yeast biocapsule methodology exploits the natural adhesion properties of yeast (i.e., biofilm formation) and filamentous fungus cells so they attach to each other thus eliminating the need of external supports and decreasing the final price of the process. Future research on the function of the *FLO11* gene as well as other genes involved in biofilm formation, in flor yeast will help boost cell-immobilization methodologies by decreasing the release of yeast cells to the external medium (Nedović et al., 2015).

García-Martínez et al. (2011) demonstrated the death of the fungus when the yeast biocapsules were incubated in media supporting yeast fermentation by effect of direct contact between its hyphae and yeast cells, and to endure as a mere, but highly inert and stable support for yeast cells, which can ease their reuse. Because of these characteristics, yeast biocapsules have already been utilized in production of white wine, sparkling wine, and natural sweet wine as well as for bioethanol from starch and molasses (Peinado et al., 2005, 2006; García-Martínez et al., 2012, 2013, 2015; Puig-Pujol et al., 2013) in a lab-scale and have been considered a promising technique for industrial-scale fermentation purposes. Comparison of white grapes juice fermentations conducted by yeast biocapsules vs. free yeasts showed higher amounts of acetaldehyde produced by the biocapsules (84 vs. 63 mg/L, respectively), isobutanol (217 vs. 194 mg/l), L-proline (7.7 vs. 6.5 mM), and aspartic acid (0.42 vs. 0 mM) in final wine. All of these analyzed compounds ranged between the limits of concentration values described in the literature and no existence of off-flavors were reported (Peinado et al., 2005). López de Lerma et al. (2012) and García-Martínez et al. (2013) used osmotolerant *S. cerevisiae* strains to form biocapsules to elaborate sweet wine from raisin must to overcome the lag phase of yeasts under osmotic stress. Fermentations resulted in high concentrations of compounds related to osmoregulation like glycerol, acetaldehyde, acetoin among others, leading to an increased complexity of wine aroma (García-Martínez et al., 2013). Biocapsule immobilization was also compared to Ca-alginate beads for sparkling wine elaboration, producing the first wine with lower calcium ion content and improved enological characteristics (Puig-Pujol et al., 2013).

In wine sensory quality analyses, results vary from different studies (Mallios et al., 2004; Tsakiris et al., 2004a,b; Genisheva et al., 2012; Gonzalez-Pombo et al., 2014). Tsakiris et al. (2004a,b) asked consumers to calculate the pleasantness of red wine samples elaborated with yeast cells immobilized and they noted scores slightly higher for immobilization although not statistically different. Knowledge about the aroma and flavor profile provided by wine yeasts combined with the utilization of mixed non-*Saccharomyces*/*Saccharomyces* starters in immobilized formats for sequential inoculations allow winemakers to use them in a scientifically controlled way to craft wine types that match consumer preferences in a diversified range of market sectors. The utilization of non-*Saccharomyces* yeast combined to *S. cerevisiae* (to avoid stuck fermentations) has been recommended to improve the quality and complexity of wine (Jolly et al., 2014; Capozzi et al., 2015). Hence, the utilization of controlled multi-starter fermentation using previously selected cultures of non-*Saccharomyces* and *S. cerevisiae* yeast strains has been encouraged (Ciani and Comitini, 2011; Comitini et al., 2011; Domizio et al., 2011; Magyar and Tóth, 2011; Di Maio et al., 2012; Morata et al., 2012; Jolly et al., 2014). Yeast cell immobilization will ease sequential inoculations of these yeasts where the selected non-*Saccharomyces* and *Saccharomyces* yeasts are in high concentrations and active; and ferment for a given amount of time one after the other until *S. cerevisiae* is added to conclude the fermentation (Canonico et al., 2016). This practice will allow the non-*Saccharomyces* yeast longer time to express their particular metabolic footprint that would otherwise be inhibited by the stress of *Saccharomyces* competition.

In the last few years, Kandylis et al. (2010), Kourkoutas et al. (2010), and Tsaousi et al. (2010, 2011) have proven the potential of elongated periods of storage for thermally dried immobilized yeast cells in different carriers (delignified brewer’s spent grains and DCM, GP, and freeze dried wheat) with neither loss of viability nor fermentation activity and making wines with similar organoleptic characteristics to those of fresh inocula, thereby accentuating the commercial potential for industrial usage. For these reasons, immobilization of microbial cells can improve cell metabolism even under stress conditions (e.g., high sugar content, low and high temperatures) and can be used for biological removal of detrimental compounds (i.e., de-acidification) or controlled liberation of flavor-active compounds, all of which improve the ability of the overall process and the quality of the end products. Indeed, the long-term storage of immobilized cells and their utilization at higher scales will boost the industrialization of immobilized technology in winemaking.

## Final Considerations

Studies evidence the advantages of immobilized yeast cells in comparison with free yeast cells. Cell immobilization has been proven to be an interesting strategy to overcome some important inconveniences in fermented alcoholic beverages production. However, though many benefits are described and new technologies are still arising, there are not many applications for winemaking at an industrial level. Potential reasons could be as follows: (i) lack of feasibility at the cellar scale – some of the methods may be difficult to up-grade, (ii) insufficient effectivity of yeast cell adherence to the carriers of current immobilization technologies, (iii) high investments (economic and time) to integrate these technologies into traditional practices without a secure outcome, (iv) lack of advertisement on immobilized yeasts, and (v) limited knowledge in winemakers about the yeast immobilization techniques and their benefits.

This review shows how studies concerning immobilization supports and matrix properties, such as their solubility, chemical and mechanical stability, their degradation in different culture broths and physico-chemical conditions during their use in a bioreactor, should be addressed more in depth. In this respect, the spontaneous and inter-species biological co-immobilization system between a GRAS fungus and an industrial yeast strain open new perspectives as a new carrier for improvement of yeast immobilization systems. Furthermore, for the implementation to an industrial scale, a higher scientific knowledge is necessary regarding the influence of immobilization on the physiology of industrial yeast strains and about the metabolites excreted, especially those directly related with the sensorial attributes of the obtained beverages.

## Conclusion

We think that immobilization systems used for yeast cells provide a revolutionary perspective for the next future in production of wine and other beverages. Their use to carry out addressed and controlled fermentation processes can contribute to innovate the production technology and the design and making of new and differentiated supreme quality products for consumers. The scarceness of industrial applications that exist in the present does not mean that research on yeast immobilization techniques should be abandoned. Aversely, investigation should be boosted in order to find the right immobilization technique for the right application in winemaking in order to exploit all the potential of these promising techniques.

## Author Contributions

JM-G performed the necessary literature searches, data compilation, and wrote the manuscript. TG-M, JCM, and JM coordinated the work and critically revised the manuscript before submission.

## Conflict of Interest Statement

The authors declare that the research was conducted in the absence of any commercial or financial relationships that could be construed as a potential conflict of interest.
